# Circadian influence on intrusive re-experiencing in trauma survivors’ daily lives

**DOI:** 10.1080/20008198.2021.1899617

**Published:** 2022-03-09

**Authors:** Alex Rosi-Andersen, Laura Meister, Belinda Graham, Steven Brown, Richard Bryant, Anke Ehlers, Birgit Kleim

**Affiliations:** a Department of Psychology, University of Zurich, Zurich, Switzerland; b Department of Psychiatry, Psychotherapy and Psychosomatics, Psychiatric University Hospital Zurich, Zurich, Switzerland; c Chronobiology and Sleep Research Group, Institute of Pharmacology and Toxicology, University of Zurich, Zurich, Switzerland; d Department of Experimental Psychology, University of Oxford, Oxford, UK; e School of Psychology, University of New South Wales, Sydney, Australia

**Keywords:** Posttraumatic stress disorder, ecological momentary assessment, intrusive re-experiencing, circadian, trauma memory, chronobiology, rhythm dysregulation, Trastorno de estrés postraumático, evaluación ecológica momentánea, re-experimentación intrusiva, Circadiano, recuerdo traumático, cronobiología, desregulación de los ritmos, 创伤后应激障碍, 生态瞬时评估, 闯入性再体验, 昼夜节律, 创伤记忆, 生物钟学, 生物钟学; 节律失调

## Abstract

**Background:**

The core clinical feature of posttraumatic stress disorder (PTSD) is recurrent re-experiencing in form of intrusive memories. While a great number of biological processes are regulated by sleep and internal biological clocks, the effect of 24-hour biological cycles, named circadian rhythm, has not been investigated in the context of intrusive memories.

**Objective:**

Here we examined effects of time of day on frequency and characteristics of intrusive re-experiencing.

**Methods:**

Fifty trauma survivors reported intrusive memories for 7 consecutive days using ecological momentary assessment in their daily life. We investigated (i) time-of-day dependent effects on frequency and distribution of intrusive re-experiencing in the overall sample as well as in PTSD versus non-PTSD and (ii) time-of-day dependent effects on the memory characteristics intrusiveness, vividness, nowness and fear.

**Results:**

Intrusive memories showed a curvilinear pattern that peaked at 2pm. Intrusive memories in the PTSD group showed a constant level of intrusive re-experiencing in the afternoon and evening, whereas a descending slope was present in the non-PTSD group. In PTSD, intrusive memories might thus be experienced in a more time-scattered fashion throughout the day, indicating chronodisruption. Intrusion characteristics did not follow this pattern.

**Conclusion:**

Although preliminary and based on a small sample size, these findings contribute to a better understanding of the everyday occurrence and characteristics of intrusive memories, and point to the added value of examining time-dependent effects, which can directly inform prevention and intervention science.

## HIGHLIGHTS

- We examined diurnal changes of frequency and distress of intrusive trauma memories in trauma survivors.Interestingly, the group with posttraumatic stress disorder (PTSD) showed a different daily pattern than the group without PTSD.
- PTSD might be associated with chronodisruption and the results point to the added value of examining time-dependent effects of intrusive memories and potentially other PTSD symptoms.

## Abstract (es)

**Antecedentes:** La característica clínica central del trastorno de estrés postraumático (TEPT) es la re-experimentación recurrente en forma de recuerdos intrusivos. Si bien un gran número de procesos biológicos están regulados por el sueño y los relojes biológicos internos, el efecto de los ciclos biológicos de 24 horas, llamado ritmo circadiano, no ha sido investigado en el contexto de los recuerdos intrusivos.

**Objetivo:** Aquí examinamos los efectos de la hora del día sobre la frecuencia y características de la re-experimentación intrusiva.

**Métodos:** Cincuenta sobrevivientes de trauma reportaron recuerdos intrusivos por 7 días consecutivos usando la evaluación ecológica momentánea en su vida diaria. Investigamos (i) los efectos dependientes de la hora del día sobre la frecuencia y distribución de los recuerdos intrusivos en la muestra general, así como en TEPT versus no TEPT y (ii) los efectos dependientes de la hora del día sobre las características de la intrusividad de los recuerdos, vividez, momento presente y miedo.

**Resultados:** Los recuerdos intrusivos mostraron un patrón curvilíneo que alcanzaba su punto máximo a las 2 pm. Los recuerdos intrusivos en el grupo con TEPT mostraron un nivel constante de re-experimentación intrusiva en la tarde y la noche, mientras que en el grupo sin TEPT se observó una pendiente descendiente. En el TEPT, los recuerdos intrusivos pueden experimentarse en una manera más dispersa en el tiempo a lo largo del día, lo que indica cronodisrupción. Las características de intrusión no siguieron este patrón.

**Conclusión:** Aunque son preliminares y se basan en un tamaño de muestra pequeño, estos hallazgos contribuyen a una mayor comprensión de la ocurrencia cotidiana y las características de los recuerdos intrusivos y apuntan al valor agregado de examinar sus efectos dependientes del tiempo, que pueden informar directamente a la ciencia de la prevención e intervención.

## Abstract (zh)

**背景:** 创伤后应激障碍(PTSD)的核心临床特征是以闯入性记忆的形式反复再体验。虽然大量的生物过程受睡眠和内部生物钟的调节,尚未在闯入性记忆的背景下研究 24 小时生物周期(称为昼夜节律)的影响。

**目的:** 这里我们考查了一天时间对闯入性再体验频率和特征的影响。

**方法 :** 50 名创伤幸存者在日常生活中使用生态瞬时评估连续 7 天报告了闯入性记忆。我们研究了 (i)对整个样本以及 PTSD 与非 PTSD 对比组中闯入性再体验的频率和分布的每日时间依赖影响,以及 (ii) 对记忆特征闯入性、生动性、当下性和恐惧的每日时间依赖影响。

**结果:** 闯入性记忆呈现出一种在下午 2 点达到峰值的曲线模式。PTSD组的闯入性记忆在下午和晚上表现出恒定水平的闯入性再体验,而非PTSD组则呈现下降趋势。因此,在 PTSD 组中,闯入性记忆可能会在一天中以更分散的方式体验,这表明时间中断。闯入特征没有遵循这种模式。

**结论:** 虽然是初步研究并且基于小样本量,这些发现有助于更好了解闯入性记忆的日常发生和特征,并指出考查其时间依赖效应的附加价值,可以直接为预防和干预提供信息科学。

## 1. Introduction

Circadian rhythms (CR) are natural, internal processes that regulate physiological activities, including the sleep-wake cycle which repeat roughly every 24 hours (Patke, Young, & Axelrod, 2019). These 24-hour rhythms are driven by a circadian clock, with a core pacemaker lying in the suprachiasmatic nucleus (SCN) (Michel & Meijer, 2020), and have been widely studied across species (Saini, Jaskolski, & Davis, 2019). Across different learning and memory measures, such diurnal and circadian rhythms have been confirmed (Smarr, Jennings, Driscoll, & Kriegsfeld, 2014). These rhythms translate to a well-established diurnal pattern for alertness which peeks around noon and late evening, whilst levels are lowest in the early morning (Beersma & Gordijn, 2007). This pattern is reflected in various neuropsychological tests (i.e. targeting attention, working memory and executive function) where performance is highest between 10:00 to 14.00 and 16:00 to 22:00 (Valdez, Ramírez, & García, 2012). Patterns in memory task performance also depend on the type and complexity of the memory at play, however (Blatter & Cajochen, 2007). In his now classic experiments, Nathaniel Kleitman documented time-of-day (TOD) effects on cognitive performance, where performance in a cognitive demanding memory task, for instance, showed patterns different from alertness with highest recall in the early afternoon (Kleitman, 1933).

The finding that learning and memory phenomena are modulated by CR has vital implications for the understanding, prevention and treatment of posttraumatic stress disorder (PTSD). PTSD has been described as a disorder of memory, with re-experiencing of intrusive emotional trauma memories as the core symptom (Brewin, 2015; Brewin, Gregory, Lipton, & Burgess, 2010). Intrusive memories are typically experienced as involuntary, brief image-based recollections of a specific autobiographical event (Brewin et al., 2010; Kleim, Graham, Bryant, & Ehlers, 2013). They are triggered involuntarily by environmental or internal cues reminiscent of the original trauma but without retrieval of the appropriate autobiographical context (Brewin et al., 2010). Intrusive memories of PTSD patients are often intense, vivid and accompanied by a sense of current threat and a sense of ‘nowness’ (Ehlers & Clark, 2000; Michael, Ehlers, Halligan, & Clark, 2005).

Recent evidence indeed suggests significant associations between disruption in CR and mental health, including PTSD (Agorastos, Kellner, Baker, & Otte, 2014; Olff et al., 2019), and studies have begun to investigate interactions between the circadian system and mental health (Walker, Walton, DeVries, & Nelson, 2020). For example, more fragmented sleep patterns after trauma have been observed in those who develop PTSD (e.g. Tsanas, Woodward, & Ehlers, 2020) and, reversely, sleep had a protective role on the development of intrusive memories (Iyadurai et al., 2019; Kleim, Wysokowsky, Schmid, Seifritz, & Rasch, 2016). Although an increasing amount of research thus suggests associations between activity dysregulation (e.g. sleep disturbances), cognitive resources, and intrusive memories, TOD dependent effects on intrusive memories in real life have not yet been investigated. Such a link could be meaningfully exploited for memory modulation and optimizing current psychotherapy for PTSD. Here we analysed data from a daily diary study on intrusive re-experiencing in trauma survivors with and without PTSD to assess whether TOD dependent effects on intrusive re-experiencing can be observed and are associated with frequency and characteristics of intrusive re-experiencing.

## 2. Materials and methods

The sample, obtained from a prior investigation, consisted of 50 survivors of assault or road traffic accidents (RTA) that had been recruited for a study capturing intrusive re-experiencing by using ecological momentary assessment (EMA) (see Kleim et al., 2013 for a more detailed description). The protocol was approved by the local ethics review board. EMA involved repeated real-time sampling of intrusive memories in trauma survivors’ naturalistic environment over seven consecutive days which made it less vulnerable to recall bias (Shiffman, Stone, & Hufford, 2008).

Intrusive memories were defined as memories of the trauma that popped to mind involuntarily. In an interview, presence and content of intrusive memories were identified using an adaptation of the Intrusion Questionnaire by Hackmann, Ehlers, Speckens, and Clark (2004). Participants were then instructed to record every intrusive trauma memory throughout their waking day for the coming 7 days. Intrusive memories were assessed with electronic diaries that allowed for detailed assessment of time of occurrence and content. Further, characteristics of intrusive memories, such as “intrusiveness, ‘nowness’, ‘vividness’ and ‘fear’ were rated by participants on a scale from 0 to 100. To limit the burden of recording, entries were restricted to one entry per hour. Additionally, the Beck Depression Inventory (BDI), a well-established self-report questionnaire, was used to assess symptoms of depression (Beck & Steer, 1987). To assess PTSD symptoms and diagnosis, an established standard semi-structured interview, the Clinician-administered PTSD scale (CAPS), was used (Blake et al., 1995). According to the reported symptoms in the CAPS and the number of symptoms specified in DSM-IV for PTSD, the sample was divided into a PTSD group (*n* = 21) and a non-PTSD group (*n* = 29). Participants with PTSD did not significantly differ from those without PTSD on any of the sociodemographic variables, *p* > 0.05 (Table 1), except on alcohol consumption which was higher in the non-PTSD group, *p* = 0.01). Logistic multilevel regressions did not indicate any moderation on the effect of time by alcohol on intrusive memory frequency (Z = −1.48, *p* = 0.14). CAPS and BDI scores were higher in the PTSD group (*p* < 0.001). More RTA survivors fulfilled diagnostic criteria for PTSD compared to assault survivors, this difference was not significant (Chi^2^ = 2.00, *p* = 0.16).

**Table 1.** Demographic and clinical sample characteristics (*N* = 50)

		Non-PTSD (*N* = 29)		PTSD (*N* = 21)			
Parameter	Group	N	%	N	%	χ2	*p* Value*
Sex	Male	12	41	7	35	0.02	0.879
	Female	17	59	13	65		
Marital status	Married	2	6.9	4	20	7.71	0.052 °
	Divorced	1	3.5	4	20		
	Single	19	66	11	55		
	Long-term relationship	7	24	1	5		
Employment	Full-time	7	24	8	40	3.30	0.653
	Part-time	5	17	4	20		
	Unemployed	10	35	5	25		
	Retired	2	6.9	2	10		
	Student	3	10	1	5		
Drug use	Never	28	97	17	85	0.36	0.357
	Occasionally/more	1	0.4	3	15		
Alcohol use	Never	5	17	11	55	0.01	0.014*
	Occasionally/more	24	83	9	45		
Medication	No Medication	20	69	15	71		
	Sleep medication	2	7	0	0	12.00	0.213
	Antidepressants	1	4	4	19		
	Other (heart, diabetes, etc)	6	20	2	10		
Type of trauma	Traffic Accident	16	50	11	57.9	2.00	0.157
	Assault	16	50	8	42.1		
		Mean	SD	Mean	SD	*t* Value	*p* Value
Age		37.8	14	38.0	18	−0.60	0.943
Time since Trauma (Months)		62.1	97	68.7	100.1	0.23	0.819
BDI		9.7	8.9	25.1	10.1	−5.47	<0.001
CAPS		13.8	7.1	30.6	9.2	−10.60	<0.001

*p-values were obtained either by χ2 test for the categorical variables or by *t*-test for the continuous variables.

## 3. Data analysis

All analyses were performed in R version 3.5.1. Distribution of intrusive memories across the seven days was established by binning the raw data into 1-hour bins. Data were summarized for visualization using a density plot, which comprised the sum of all observations occurring within a time of the day in the overall sample, and an average score plot, which comprised the average number of intrusive memories within individuals. Differences in sum-score distribution between non-PTSD and PTSD participants were established via a Chi-Square test. Logistic Poisson multilevel regression analysis was used to verify the effect of time of day effect on the number and characteristics of the intrusive memories. Further evidence and visualization of periodicity for intrusion frequency was gathered via the utilization of the Lomb-Scargle periodograms, a well-known algorithm for assessing periodic signals within unevenly sampled data. In view of the exploratory nature of this paper, the effects on the four memory characteristics were considered separately.

## 4. Results

### 4.1. TOD effects on frequency and distribution of intrusive memories in PTSD vs non-PTSD

**Figure 1:**
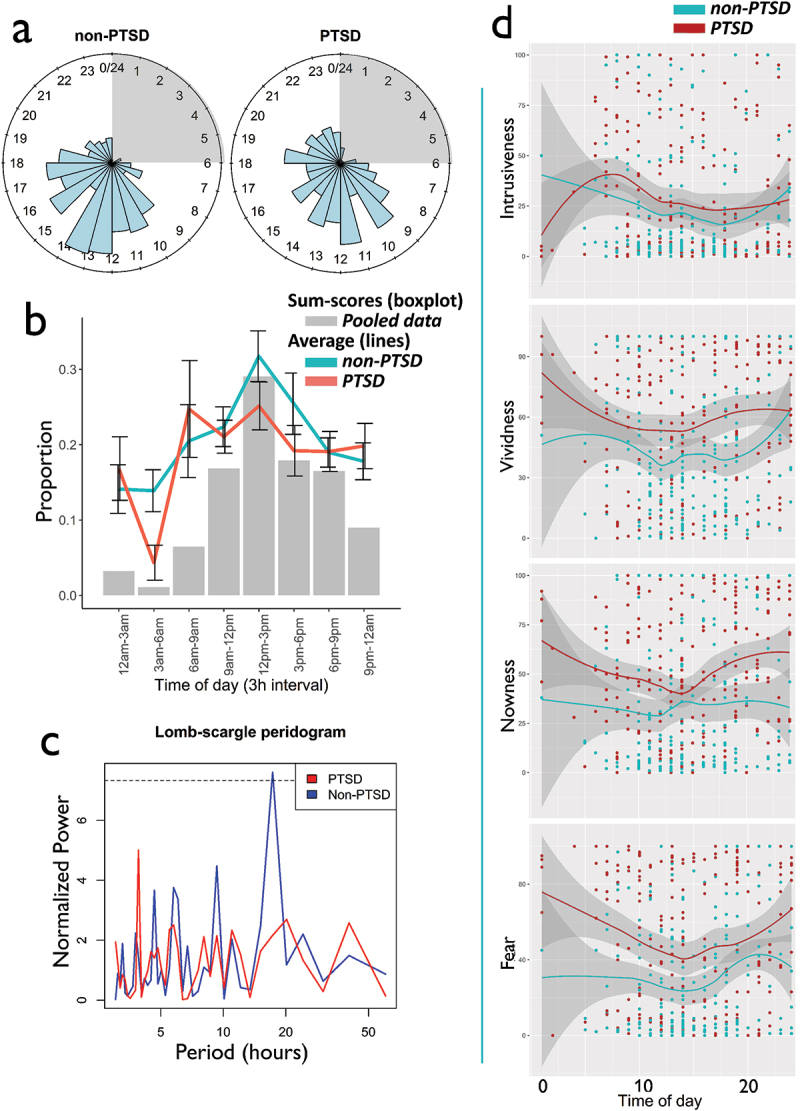
(a) Density plot (sum-scores) for non-PTSD (left) and PTSD participants (right) across the day (χ2 = 175.56, *p* = 0.03). (b) Average distribution (non-PTSD vs PTSD) vs sum-scores comparison. 3 hour bins were chosen for the representation in order to avoid overloading the figure. Average and sum-scores to the same scale, the percentage of events calculated. (c) Lomb-Scargle power-spectrum performed to create the discrete spectrum representation of our periodic signal. Normalized power is the result obtained from the Fourier transform of its auto-correlation signal. (d) Non-linear regression for Intrusiveness, Vividness, Nowness and Fear.

**Figure 2.**
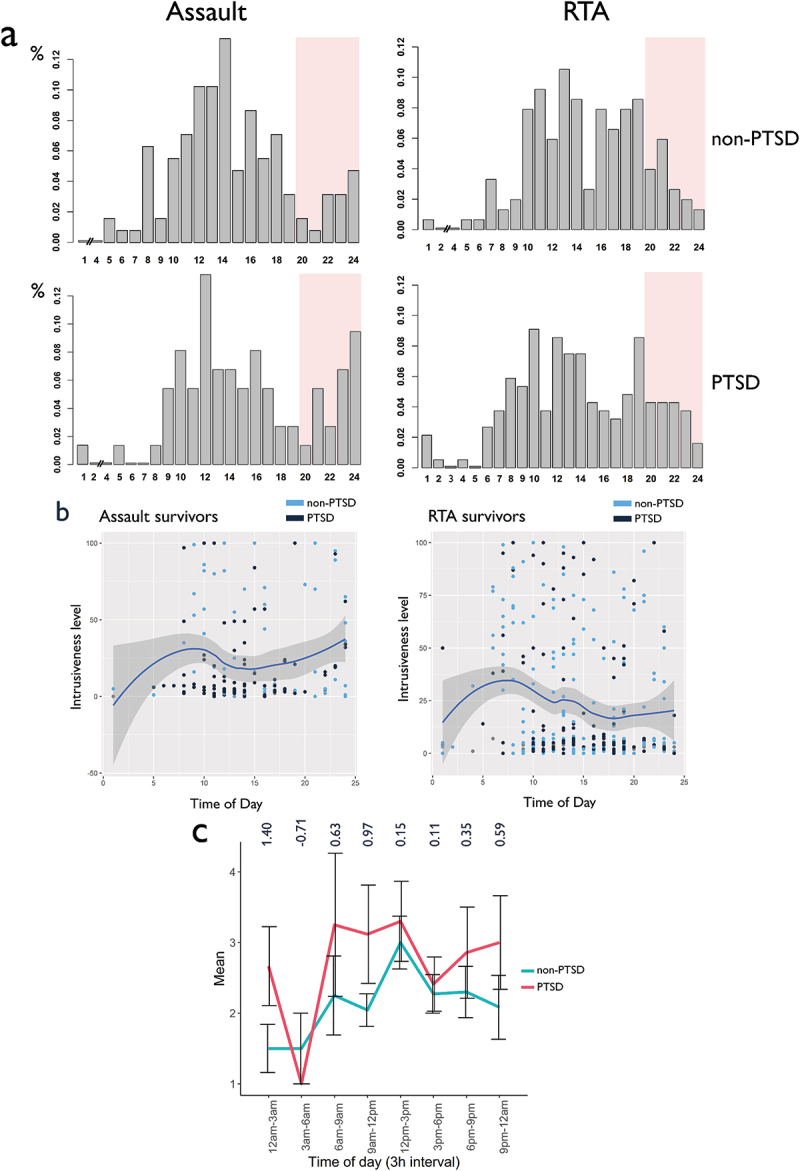
(a) Density plot (sum-scores) for non-PTSD (upper panel) and PTSD participants (lower panel) across the day subdivided by type of trauma (Assault left, RTA right). Evening period highlighted by red squares. (b) Non-linear regression for Intrusiveness in Assault and RTA. All individuals were pooled for this graph as effects were deemed similar in PTSD and non-PTSD groups. (c) Average distribution of the raw number of reported intrusion memories (non-PTSD vs PTSD) throughout the day. 3-hour bins were chosen for the representation in order to avoid overloading the figure. Values above the 3-hour averages represent effect sizes of PTSD for that specific time bin. Effect sizes were computed regardless of significance.

**Figure 3.**
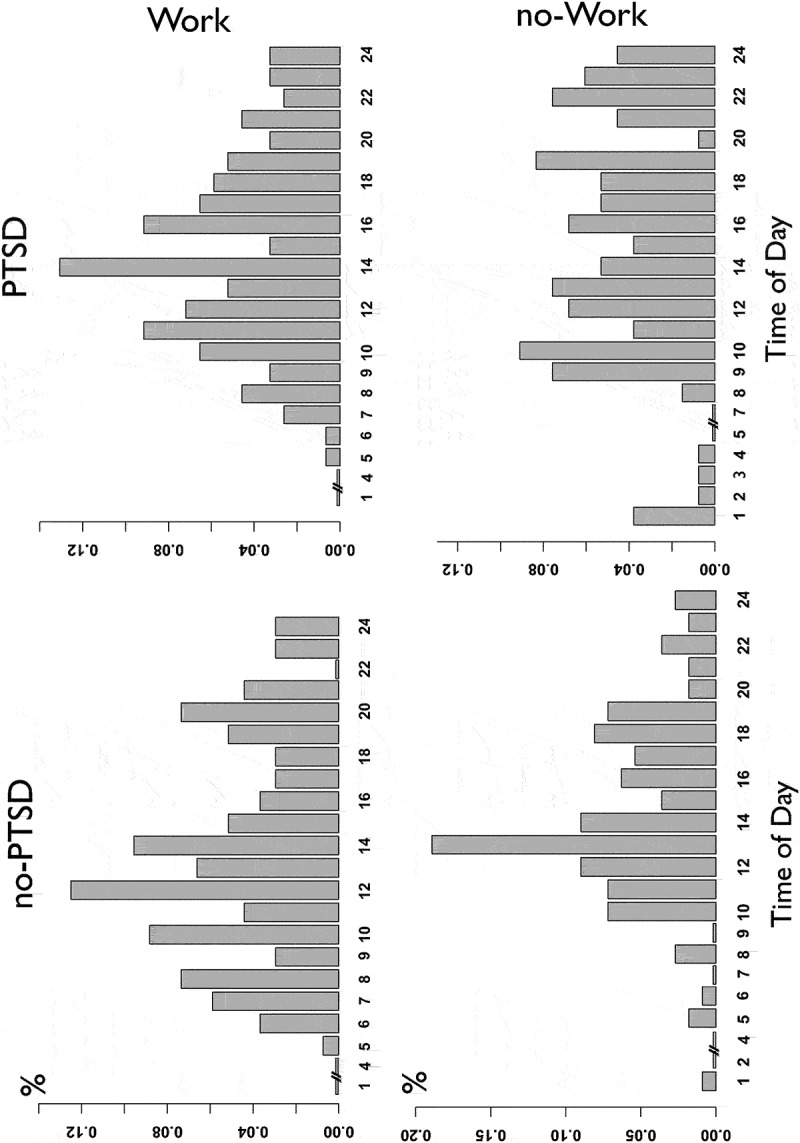
Density plots (sum-scores) for non-PTSD (left) and PTSD participants (right) across the day for patients who are currently working (top row) versus those who are unemployed (lower row). Periodicity appears visually absent from the unemployed PTSD group with the loss of rhythmicity between noon and evening (mean noon = 7.6, mean evening = 7). However, following the subdivision of our data in four groups, signi-ficant periodicity could only be confirmed with a Lomb-Scargle periodogram in the unemployed non-PTSD group (power = 7.3, *p* = 0.026).

PTSD participants reported a significantly higher number of intrusive memories compared to non-PTSD participants, *Mean*_PTSD_ = 14.21, SD ± 10.8 (Median_PTSD_ = 15, MAD = 7.40), *Mean*_Non-PTSD_ = 10.27 (Median_Non_PTSD_ = 12, MAD = 5.83), SD ± 6.83, *t* = 2.72, *p* = 0.007. Eight individuals from the PTSD group reported intrusions during night-time, while only four individuals did in the non-PTSD group. This difference was not significant (*t* = 1.74, *p* > 0.05). To prevent artefacts in the frequency analysis due to the low number of night-time observations (12pm-6am), these were removed from any analyses assessing the presence of periodicity (Figure 1(a)). Frequency of intrusive memories was found to fluctuate significantly (z < 2.75, *p* < 0.001) in both the PTSD and non-PTSD groups based on the time of day (Figure 1(a), Table 2b). Distributions observed on 24-h density plots differed significantly between the non-PTSD and PTSD groups (*χ^2^ *= 175.56, *p* = 0.03). While both distributions rapidly peaked between 12pm and 2pm and progressively decreased towards the evening (Figure 1(a)), the PTSD group’s peak appeared shifted towards the morning and evening re-experiencing was more elevated (*t* = −3.16, *p* < 0.05). Direct comparison of re-experiencing frequency distributions obtained by sum-scores and within-individuals average indicated similar findings with a peak occurring around noon. However, the average distribution had proportionally higher baseline levels in the morning and evening as compared to the density plot (Figure 1(b)). Further confirmation of significant group differences was provided by a Granger test on the raw time-series (F = 2.51, *p* = 0.12). To investigate the potential impact of PTSD at different times of days on the number of intrusions, we also assessed the effect size of group (PTSD versus Non-PTSD) at different time points of the simple mean distribution (Figure 2(c)). Effect sizes fluctuated between 0.1 and 0.9 depending on the time of day. Larger effects for PTSD were found in the morning and afternoon. Unemployment was found to impact the effect of TOD on intrusive memory frequency (Fisher’s *p* < 0.001) in the overall sample, with more time-scattered reports of intrusive memories throughout the day (Figure 3) in those unemployed, but no differences between participants with and without PTSD and PTSD (Fisher’s *p* = 0.62), in those employed.

**Table 2.** Logistic poisson multilevel regressions

a. Regression model predicting the amount of intrusive memories based on time of day (random effect = Individuals)							
Group	Beta	Sd. error	Z-value	Rand. effect	*p*-Value		
Non-PTSD	−0.046	0.013	−3.676	0.192	<0.001		
PTSD	−0.035	0.013	−2.757	0.273	<0.001		
b. Regression model predicting the relationship between single characteristics and time of day (random effect = Individuals). PTSD diagnosis interaction was controlled for (interaction) in the model and removed if the interaction was non-significant.							
Predicted	Interaction	Predictor	Beta	Sd. error	df	*t*-Value	*p*-Value
Intrusiveness	No	Time of day	−0.536	0.231	372	−2.323	0.02*
Vividness	No		0.286	0.247	362	1.157	0.248
Nowness	No		0.295	0.247	362	1.183	0.237
Fear	No		0.134	0.28	351	0.48	0.63

When performing Lomb-Scargle periodogram analyses (Figure 1(d)), comparisons indicated the presence of a significant 24-hour periodicity (PNmax = 7.33, *p* = 0.03) in the non-PTSD group. A 24-hour period was absent from the PTSD group, when sleep effects were excluded (PNmax = 4.95, *p* = 0.39).

We assessed whether type of trauma (RTA, assault) had an impact on frequency and characteristics of intrusions. The comparison of RTA vs assault survivors by Poisson multilevel regressions yielded significant (non-PTSD group: Z = 2.32, *p* < 0.05) or marginally significant (PTSD group: Z = 1.65, *p* < 0.1) differences in the frequency of intrusions during the evening. The frequency of intrusions was significantly lower during the evening in RTA survivors while it peaked again after 9pm in assault survivors (Figure 2(a)). The same distribution (Figure 2(b)) was also supported for intrusiveness levels (Z = −2.41, *p* < 0.05).

### 4.2. TOD effects on intrusive memory characteristics in PTSD and non-PTSD

Our dataset contained a total of 31 night-time intrusions reported by 16 participants. Compared to day-time intrusions within the same individuals, these night-time intrusions were accompanied by greater fear (*t* = 2.63, *p* = 0.01), vividness (*t* = 2.17, *p* = 0.04) and feelings of helplessness (*t* = 2.13, *p* = 0.04). Due to the very low number, and to prevent artefacts in assessing the presence of a signal throughout the day, we excluded these 31 observations from the following Logistic Poisson multilevel-regressions. The inclusion or exclusion of these observations did not alter signi-ficance of the respective results.

Results from logistic Poisson multilevel-regression on characteristics of intrusive memories did not reveal significant differences for the effect of time on intrusiveness (*t* = 1.15, *p* = 0.25), vividness (*t* = −0.24, *p* = 0.81), nowness (*t* = 0.89, *p* = 0.37) and fear (*t* = −1.44, *p* = 0.15) between individuals with and without PTSD (Table 2b). We thus pooled non-PTSD and PTSD participants when assessing the effect of time alone on the distribution. Analysis on the pooled group found a significant effect of time of day on intrusiveness (*t* = – 2.06, *p* = 0.04). This effect was not observable for vividness, nowness and fear (Table 2b). Based on the Lomb-Scargle periodograms, we did, however, find 24-h periods for nowness (PNmax = 6.81, *p* = 0.04) and fear (PNmax = 7.91, *p* = 0.01), a marginal effect for intrusiveness (PNmax = 5.64, *p* = 0.12) and no effect for vividness (PNmax = 4.16, *p* = 0.45). Visual observation of plotted linear regressions (Figure 3(d)) suggested that all characteristics of intrusive memories showed greater scores during morning and evening as compared to the rest of the active day, with a minimum reached between 12pm and 4pm. This general pattern was also observable in vividness.

## 5. Discussion

PTSD has been associated with the disruption of daily rhythmic activity, and some have even suggested that evaluation and treatment of such disruption and sleep should be the first steps in PTSD management (Agorastos et al., 2014). To the best of our knowledge, this is one of the first studies to investigate diurnal patterns of frequency and characteristics of intrusive memories in survivors of trauma with and without PTSD. Investigating daily diaries in trauma survivors for seven consecutive days, we identified a rhythmic pattern in the frequency of intrusive memories. Intrusive memories showed a curvilinear pattern that peaked around 2 pm. Supporting previous findings, intrusive memory frequency and their characteristics were significantly higher in participants with PTSD compared to those without PTSD and significant differences between the daily temporal patterns of intrusion frequency were present when comparing these two groups. Both sum and mean score distributions for participants with PTSD showed a heightened constant level of intrusive re-experiencing in the afternoon and the evening, whereas a descending slope was present in non-PTSD participants. Furthermore, the time of highest frequency for intrusive memories was shifted towards the morning in the PTSD group which might be associated with sleep disruptions including sleep problems in the early hours of the day in the PTSD group (De Boer et al., 2020; Germain, 2013). The results suggest that participants with PTSD might experience intrusive memories in a more time-scattered fashion and more persistently throughout the day. This aligns with research stating that PTSD is associated with chronodisruption (Agorastos et al., 2014) and points to the added value of examining its time-dependent effects.

There is significant room for improving current PTSD treatment (Lewis, Roberts, Andrew, Starling, & Bisson, 2020) and identification of TOD dependent patterns may be useful to help optimize treatment options. Potential applications would be identification of optimal times-of-day for psychotherapeutic interventions. Teaching patients behavioural strategies to manage intrusive memories and patients recruitment of such strategies at specific TOD might be particularly effective, as well as pharmacological treatment options targeting trauma memories and their implementation based on diurnal temporal patterns of occurrence of intrusive memories (Roenneberg & Merrow, 2016). Such optimization or personalization of treatment effects based on basic science has been called for in order to improve existing treatment options (Holmes, Craske, & Graybiel, 2014).

The identified pattern for intrusive re-experiencing in our study is inconsistent with the well-established shape of alertness and psychomotor patterns found in previous studies of arousal and attention (Beersma & Gordijn, 2007; Valdez et al., 2012), thereby suggesting that the distribution we observe in this study does not stem from the well-known interaction of sleep and circadian processes, but from other underlying mechanisms. Comparing our pattern of intrusive memories with known patterns of performance in memory tests, we found that it was best linked to the performance in complex memory tasks found in Kleitman (1933). Our findings could be in line with the understanding that complex emotional learning and disrupted contextual modulation of fear is often implicated in PTSD formation which could be reflected by intrusive memory recall (Colvonen, Straus, Acheson, & Gehrman, 2019). Another way to interpret the pattern is to focus on intrusive memories as involuntary memories different from the voluntary recalls formerly examined in the assessment of diurnal patterns. Intrusive memories occur more often when the ability to suppress their retrieval is low (Streb, Mecklinger, Anderson, Lass-Hennemann, & Michael, 2016). This ability is a part of executive functioning and could explain that in our study frequency of intrusive memories was high when executive functioning was supposed to be low (mornings, early afternoon and later at night).

Density plots varied significantly between PTSD and non-PTSD participants, while the difference appeared as a trend in average-based distributions. This may indicate that the results were mainly driven by those participants affected by more intrusive memories. However, providing partial focus on individuals with a high intrusive memory frequency appeared useful in the current context, as findings may help understand the dynamics experienced by those trauma survivors, and PTSD patients, that are most heavily affected by re-experiencing symptoms.

No significant interaction between TOD and PTSD was found for intrusion characteristics. The investigation of the pooled data from PTSD and non-PTSD populations uncovered some trends in the presence of TOD dependent patterns for memory characteristics (intrusiveness, vividness, nowness and fear). These trends favoured the notion that trauma survivors experience intrusions as more burdensome during morning and evening hours. This may indicate highest emotional response to intrusive memories at the time of heightened re-experiencing in PTSD. The finding that participants with PTSD experience continuously heightened levels of intrusions in the evening and a morning-shifted frequency peak could be particularly relevant. Sleep disturbances, which are likely to be associated with experiencing intrusive memories at these times, have been reliably identified as a risk factor for suicidal ideation (Perlis et al., 2016). Experiencing intrusive trauma memories at night may confer increased risk, as they may lead to additional distress and decrease the likelihood of engaging in adaptive cognitive and behavioural strategies. The finding that intrusive memories’ peaks were often reached during morning hour in PTSD could thus be relevant for designing effective prevention and intervention strategies.

Finally, the data indicated differences in daily intrusion patterns between types of trauma. Intrusion frequency and intrusiveness dropped in RTA survivors during the evening whereas assault survivors experienced a peak towards the late evening. While this study was not designed to enable the comparison of recall time to the time of trauma, the fact that most intrusions occur during the daytime for RTA survivors when people are more likely to travel and traffic is busier points to the role of trauma triggers. The evening peak in assault survivors may be related to worry about intruders. The findings provide some potential hypotheses about the importance of surrounding cues rather than the time of recall as such. The impact of specific types of trauma on the time of re-experiencing is interesting and should be investigated by future studies.

There are several limitations associated to the presented dataset. Despite various advantages of EMA, its sampling method led to a variability in numbers of reported memories, therefore creating large heterogeneity. This may have led to low power for some analyses and thus inability to detect some of the effects in a heterogenous sample. Most diary studies to date are comparably small and future studies should index intrusive memories in individuals’ daily lives in larger sample sizes. The diary-based sampling method did not allow for consistent collection of intrusions during night-time and we might have not captured night-time intrusions in some of our participants. Participants were not studied under a constant condition, such as sleep deprivation, thereby excluding the possibility to fully confirm our hypothesis of real circadian rhythms related to the biological clock. Our study also did not include a sleep diary which would have been useful in assessing observations pertained to wake behaviour. The exclusion of observations occurring from 12am to 6am is likely to have integrated some episodes linked to sleeping or wake hours incorrectly. However, as the main focus of the study was to assess the diurnal distribution of intrusions, we believe that the effect of these errors to be limited. Furthermore, we did not control for other time-dependent effects. For example, Boland and colleagues (Boland et al., 2019) hypothesized that social rhythm dysregulation might play a role in survivors of trauma. Relatedly, experiencing intrusive memories depends on exposure to external and internal cues (Brewin et al., 2010). Depending on trauma, individuals might, at certain times of the day, be more likely to be exposed to external cues. RTA survivors could, for instance, experience intrusive memories more frequently on their way to work, thereby providing a potential explanation for an earlier time for the peak intrusion frequency. This could also explain differences in some of the patterns depending on employment. Finally, entries were restricted to one intrusive memory per hour, to reduce participants’ recording burden, which could have attenuated the circadian pattern. Additional and larger studies are therefore warranted. Such studies could also investigate work and social rhythms more systematically.

In conclusion, our results suggested the presence of circadian patterns in re-experiencing in trauma survivors’ everyday lives. The finding that intrusive memories are more common at a specific time of day could inform future studies involving time-dependent assessments. Further understanding of circadian rhythm effects in PTSD could lead to a better understanding of PTSD and other disorders characterized by intrusive emotional memories. Such results could also inform innovative treatment approaches. PTSD treatment modules that target intrusive memories could take into account circadian variation in intrusion occurrence and corresponding situational and behavioural patterns. Discrimination of intrusion triggers, for instance, which is employed in trauma-focused cognitive therapy (e.g. Wild et al., 2020), might be most critical and relevant when intrusions are most prevalent, i.e. in the evening. If more evidence is accumulated supporting circadian rhythms in PTSD, then evidence-based PTSD treatment could be tailored specifically and delivered during certain times of day. It might also open the road to new treatment approaches attempting to normalize circadian rhythms in trauma survivors with PTSD.

## Data Availability

Data are not made publicly available due to participants’ privacy and to be in accord with the original ethics proposal approved by the local ethics committee. Anonymized data will nevertheless be available from the corresponding author birgit.kleim@uzh.ch.
